# Overcoming COVID-19 vaccination resistance when alternative policies affect the dynamics of conformism, social norms, and crowding out

**DOI:** 10.1073/pnas.2104912118

**Published:** 2021-06-07

**Authors:** Katrin Schmelz, Samuel Bowles

**Affiliations:** ^a^Department of Economics, University of Konstanz, 78457 Konstanz, Germany;; ^b^Thurgau Institute of Economics (TWI), 8280 Kreuzlingen, Switzerland;; ^c^Behavioral Sciences Program Santa Fe Institute, Santa Fe, NM 87501

**Keywords:** endogenous preferences, crowding out intrinsic motivation, trust, policy implementation, state capacities

## Abstract

What is an effective vaccination policy to end the COVID-19 pandemic? We address this question in a model of the dynamics of policy effectiveness drawing upon the results of a large panel survey implemented in Germany during the first and second waves of the pandemic. We observe increased opposition to vaccinations were they to be legally required. In contrast, for voluntary vaccinations, there was higher and undiminished support. We find that public distrust undermines vaccine acceptance, and is associated with a belief that the vaccine is ineffective and, if enforced, compromises individual freedom. We model how the willingness to be vaccinated may vary over time in response to the fraction of the population already vaccinated and whether vaccination has occurred voluntarily or not. A negative effect of enforcement on vaccine acceptance (of the magnitude observed in our panel or even considerably smaller) could result in a large increase in the numbers that would have to be vaccinated unwillingly in order to reach a herd-immunity target. Costly errors may be avoided if policy makers understand that citizens’ preferences are not fixed but will be affected both by the crowding-out effect of enforcement and by conformism. Our findings have broad policy applicability beyond COVID-19 to cases in which voluntary citizen compliance is essential because state capacities are limited and because effectiveness may depend on the ways that the policies themselves alter citizens’ beliefs and preferences.

## Significance

We provide a model of policy effectiveness to explore the dynamics of vaccine resistance, drawing on our panel data set. The key ideas motivating the model are that voluntary citizen compliance is essential to policy success even under enforcement and that compliance preferences are endogenous, possibly crowded out by enforcement or enhanced due to conformism as more other citizens comply. Our panel data tracks intraindividual changes in trust in public institutions and vaccine acceptance, allowing inferences about causal effects. Our contribution is the integration of three features: 1) a model of interaction of public policy and citizen preferences, 2) using appropriate data, and 3) allowing insights on how to address the COVID-19 pandemic and other important societal challenges.

Legally required vaccination against measles and other diseases is an essential part of public health policies around the world. But opposition to even voluntary COVID-19 vaccination has emerged in many countries. During the initial rollout of vaccinations in the United States, for example, an antivaccine demonstration temporarily closed down one of the country’s largest vaccination sites (1).

In early March 2021, among Americans not already vaccinated, 38% said that they would not willingly do so (2). In late March, among rural Americans who were not yet vaccinated, only a quarter were willing to be “as soon as possible” (3). In mid-February, a *Wall Street Journal* opinion piece authored by two former heads of the US Food and Drug Administration warned of a “vaccine glut.” By April, “[t]he challenge won’t be how to ration a scarce resource, but how to reach patients reluctant to get vaccinated” (4).

Perhaps in response to concerns that voluntary vaccine take-up would be insufficient to end the pandemic, in all but 3 of the 14 countries surveyed, majorities of those expressing an opinion supported mandatory vaccination policies (this was in late January, prior to the discovery of rare adverse effects of the AstraZeneca vaccine) (5). In March, the government of Galicia in Spain announced that vaccinations would be mandatory with violations subject to substantial fines (6). In April, Italy made vaccinations mandatory for health care workers (7). At the same time, the Chinese government ordered local authorities to cease mandatory vaccines, fearing adverse public reaction (8). In the United States, 41% of those surveyed in March said they were “very concerned” that they “might be required to get the COVID-19 vaccine even if [they] don’t want to,” and another 21% were “somewhat concerned” (9).

An important fact motivating our evaluation of policies to overcome resistance to vaccination is that those hesitating appear to be taking their cues from others. In late February, 27% of Americans not already vaccinated said they would “wait […] to see how it is working for other people.” A total of 69% of those in households in which someone had already been vaccinated also wanted to be vaccinated “as soon as possible,” while this was true of only 37% of those who do not know anyone vaccinated (10).

Our panel data set from Germany allows us to ask how vaccine resistance is changing and to identify some of the determinants of these changes, including changes in public trust and whether vaccination is voluntary or legally mandated. To apply our findings to anti–COVID-19 policy making, we develop a model of the dynamics of vaccine resistance. This model illustrates how the willingness to be vaccinated may vary over time in response to the fraction of the population already vaccinated and whether this has occurred voluntarily or not.

## Results

### With Infections Rising, People Withdrew Support for Enforced Vaccinations: Panel Evidence.

Our panel of 2,653 Germans, surveyed both in April/May and in October/November of 2020, allows us to track the attitudes of the same individuals during the first and second lockdowns in Germany (see the *Methods*). The German government’s explicit endorsement of voluntary vaccination regimes has not changed over the course of the two waves of the survey (for details, see the timeline in *SI Appendix*).

**Fig. 1.**
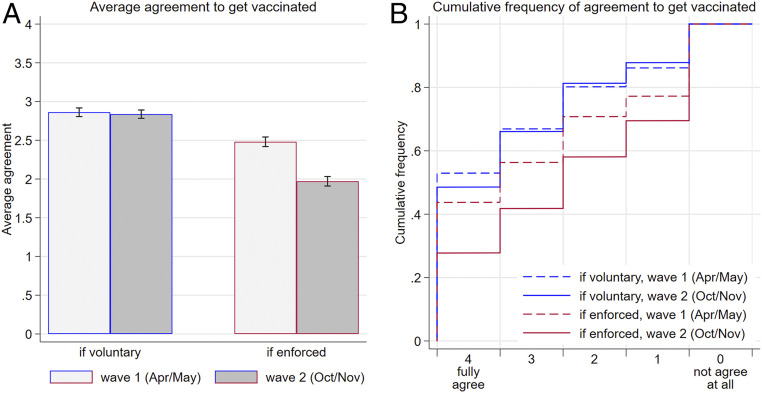
Reduced support for enforced vaccination. (*A*) Average agreement to get vaccinated if it is voluntary or enforced in the two waves of the survey (in Likert scale units). Error bars represent 95% CI. (*B*) Cumulative distributions of agreement in case of enforced versus voluntary vaccination for the two waves of the survey. For example, the dashed and solid red lines show that 44% and 28% of respondents fully agreed to get vaccinated in case of enforcement in the first and second waves of the survey, respectively. The sum of those expressing either agreement level 3 or 4 under enforcement amounts to 56% in wave 1 and 42% in wave 2. Opposition to enforcement (levels 0 and 1) was expressed by 29% in wave 1 and 42% in wave 2 (1–0.71 and 1–0.58, respectively, that is, the final two steps in the graph).

In both waves, respondents were asked: “If there is an approved vaccine against the coronavirus: To what extent would you agree to be inoculated yourself if: … vaccination is strongly recommended by the government but remains voluntary? … vaccination is made mandatory and checked by the government?” Answers were given on a five-point Likert scale ranging from 0 (“not agree at all”) to 4 (“fully agree”). While support for voluntary vaccination remained virtually unchanged in the second wave [replicating the results reported in (11)], the fraction fully supporting enforced vaccinations dropped from 44% to 28%, as shown in Fig. 1 *A* and *B*.

A total of 40% of those who had earlier agreed fully or mostly to get vaccinated in case vaccinations were legally enforced withdrew their support for the policy. Just 18% who had previously not supported enforcement became supporters. The numbers of those who did “not agree at all” to get vaccinated in case of enforcement increased from 23% to 30%. A total of 38% of the respondents reduced the degree of their support for a policy of enforced vaccines between the two waves of the survey, while only 15% increased support. The changes in a given individual’s responses over time are also illustrated in *SI Appendix*, Fig. S4.

### Determinants of Vaccination Hesitancy.

**Fig. 2.**
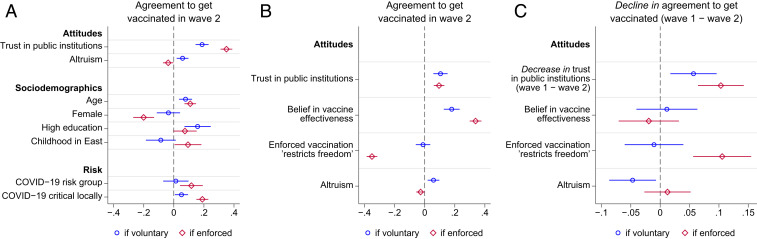
Predictors of support for voluntary and enforced vaccinations in the second wave of the survey (*A* and *B*) and of changes in support between the two waves (*C*). Shown are the coefficients and 95% CI, estimated in ordinary least squares linear regressions with standardized variables (*SI Appendix*, Tables S3 and S4). The regression model for *B* is identical to the one for *A*, except that the two variables on effectiveness and freedom are included in *B*. *C* shows that a standard deviation (SD) decrease in public trust between the two waves is associated with a decline in support for enforced vaccination of 10% of a SD and a decline in support for voluntary vaccination of 5% of a SD. These estimates are unchanged if the effectiveness and freedom variables are excluded. Note that the scale of the horizontal axis is the same in *A* and *B* but differs for *C*. The two questions concerning attitudes towards the vaccine were asked only in the second wave.

Predictors of agreement to get vaccinated and its change are shown in Fig. 2. Before exploring the mechanisms behind these changes, we first look at predictors of agreement to be vaccinated in the second wave (Fig. 2*A*). Support for enforced vaccinations in October/November 2020 was much more likely to be expressed by those who believed that the pandemic was critical locally. This makes withdrawal of support for government-mandated vaccination particularly striking, given that between the two waves of the survey, the daily infection rate in Germany increased 15-fold from an average of 1,100 new daily cases with case rates falling to an average of about 16,500 with case rates rising (*SI Appendix*, Fig. S3).

Women were much less likely to agree if vaccination was legally required but not if voluntary. Those born in East Germany were more likely to support enforced vaccination and less likely to support vaccination if voluntary.

The more respondents trust public institutions, the more they agree to get vaccinated under both implementation schemes. Our indicator of an individual’s trust in public institutions is the average of their expressed general trust in the federal government and specific trust in its truthful information about COVID-19 as well as their trust in the state government, in science, and in media (which are all highly correlated, see *SI Appendix*, Table S2). We term this measure “public trust.”

Public trust has the largest estimated (normalized) effect for support of both voluntary and enforced vaccination in Fig. 2*A*. One standard deviation (SD) difference in public support is associated with over one-third of a SD difference in support under a policy of enforced vaccination. The effect is smaller for voluntary vaccinations but still substantial.

To understand the mechanisms that might explain why distrust of public institutions is associated with opposition to vaccinations, we include measures of respondents’ beliefs that enforced vaccination restricts their freedom and that the vaccine is effective, as shown in Fig. 2*B*. The individual’s belief that the vaccine is effective in containing the virus covaries strongly with their support for enforced vaccination and also, though less strongly, for voluntary vaccination. The perception that enforced vaccination restricts a respondent’s freedom is associated with a large reduction in support for enforced vaccination but not for voluntary vaccination. The much-reduced effect size for public trust in Fig. 2*B* (compared to Fig. 2*A*) shows that a substantial portion of the statistical association of trust and vaccine support is accounted for by the fact that those who distrust public institutions are more likely to believe that the vaccine is not effective and that if mandated, it restricts their freedom.

One cannot infer causal relationships from Fig. 2 *A* and *B* because they are based on data from just one cross-section at a given point in time in our panel data. The statistical associations reported could be due to the unobserved differences among individuals. The covariation of distrust and vaccine hesitancy, for example, could arise because both are the consequence of the individual having been raised to feel antipathy toward anyone beyond their immediate family. However, our panel data allow us to map a given individual’s changes in public trust and their associated changes in vaccine support, eliminating the confounding effect of such time-invariant unobserved individual characteristics.

We find that the fall in support for enforced vaccination occurred disproportionately among those whose public trust declined between the two waves of the survey (Fig. 2*C*), suggesting that the association between trust and vaccine support frequently reported in cross-section data (11, 12) may reflect a causal relationship. The distrust effect is substantial: a one-point drop in our public trust measure (ranging from 1 to 6.6) would account for 37% of the observed reduction in support for enforced vaccines between the two waves, as explained in *SI Appendix*. However, we also show in *SI Appendix* that the panel-estimated coefficients of public trust are less than one-third of the equivalent cross-section estimates (models 1 and 3 of *SI Appendix*, Tables S3 and S4), consistent with the causal effect being smaller than the statistical association in the cross-section data.

The growing opposition to enforced vaccination in Fig. 1 is not the result of an overall decline in public trust, which remained high on average (*SI Appendix*, Table S2). Nor is the withdrawal of support for enforced vaccinations the result of growing skepticism about the vaccine; instead, it appears to reflect increased opposition to enforcement per se. Support for getting vaccinated if the policy is voluntary remained high across the two waves of the survey (Fig. 1 *A* and *B*) with two-thirds expressing support—substantially more than those supporting enforced vaccination.

### Enforcement May Crowd Out Intrinsic Motivation and Reduce the Positive Effects of Conformism.

Effective vaccination campaigns can often rely on a degree of altruism among citizens (13). Altruism is important because, to be effective, vaccination is required of those who are not themselves vulnerable to serious illness but who can nonetheless be effective transmitters (14).

The greater support for voluntary as opposed to enforced vaccinations is consistent with the idea that intrinsic or social motivations associated with getting vaccinated may be diminished by removing the vaccination decision from an individual’s choice by implementing it as a government mandate. There are three mechanisms by which this might occur (15).

The first is “psychological reactance” (16) or what economists term “control aversion” (17), a particular case of intrinsic motivation being crowded out by explicit constraints or incentives (18, 19). Such a response has been observed in numerous experiments and is interpreted as the result of individual strivings for freedom or “self-determination” (20, 21) as has also been found for vaccine hesitancy (22). This is consistent with our finding that the opposition to enforced vaccinations was substantially greater among respondents who reported that it would restrict their “freedom” (Fig. 2*B*).

Second is what psychologists termed the “moral disengagement” that occurs because the provision of explicit incentives or constraints frames the decision problem as one in which ethical convictions are not salient (23). Voluntary vaccination policies may trigger moral deliberation and convictions to be a good citizen, whereas enforcement might relieve the citizen of any need to deliberate and thus might crowd out those moral convictions (24).

Resistance to vaccination provides an example of moral disengagement. In the second wave of the survey, those reporting greater altruism—willingness to help others—were also more likely to support voluntary but not enforced vaccinations (Fig. 2 *A* and *B*). The negative impact of enforcement is greater among citizens reporting more altruistic preferences.

The third mechanism through which enforcement may crowd out intrinsic motivation is by diminishing trust. Citizens may interpret enforcement as evidence that the policy maker knows that the vaccine is not something citizens would willingly subject themselves to. In addition to providing “bad news” (15) about the vaccine, enforcement may also both undermine trust in the state and signal that the state does not trust the individual to respect the social norm of protecting others (25). Thus, the distrust communicated by enforcement signals low expectations about citizens’ behavior. In the eyes of citizens, this may result in a mutually distrusting relationship (26, 27) promoting vaccine hesitancy, as our panel data show.

The possibility that the implementation of a public policy may alter citizens’ beliefs and preferences in ways that may compromise the effectiveness of the intervention has been explored by economists and political scientists (24, 28–30). For example, the celebrated “Lucas critique” is directed at macroeconomic policy making that ignores these effects (31). Consistent with this reasoning, our survey evidence shows that a policy can itself adversely affect citizens’ preferences upon which the policy’s success depends. This will be the first element of our model of anti–COVID-19 policy implementation.

The second element is conformism, which we define broadly as the tendency of people to adopt some behavior, belief, or other learned trait conditionally on others having adopted it. This has been a foundational principle of human behavior studied by psychologists, who associate it with feelings of anxiety or discomfort if one differs from others, and with the effect of “mere exposure” (32–34). It has been increasingly used in modeling social norms and preferences—initially by anthropologists and subsequently also by economists (35–41). In these models, conformism is an aspect of learning from others in which the likelihood that an individual's behavior will be copied is greater the more frequent that behavior is in the population. Conformist learning rules are used by many nonhuman animals and can evolve to become prevalent under a wide range of conditions (42, 43). Finally, in many experiments—for example, public goods games—subjects are conditional cooperators who contribute only if others also do so (44).

The effects of conformism or “herd behavior” may be key to the effectiveness of public policies: A study in Russia, for example, estimated that one-third of the reduction in heavy drinking that would result from doubling the price of vodka would occur through the conformist effects of the reduction in the amount of drinking by peers (45).

Conformism can have a positive effect on the diffusion of a novel vaccination even if initial acceptance by a population is modest. This is because those initially willing to be vaccinated may send a positive signal regarding their willingness to cooperate and their belief in the vaccine’s safety and effectiveness. But complying with enforced vaccination sends a much weaker signal. A possible result is that enforcement not only crowds out pro-vaccination motivation but also dampens positive conformism-based effects of others having been vaccinated.

### A Dynamic Model of Citizens’ Vaccination Preferences.

We thus set aside models of policy implementation in which citizens’ beliefs and preferences are taken as given. Instead, we show that our survey results can be applied to public policy using our model, in which citizens’ attitudes toward vaccination change over time as a result of the nature of the policy introduced and the fraction of citizens already vaccinated. Specifically, we will illustrate how a modest increase in the level of public trust or the decision to legally require vaccination could fundamentally alter the dynamics of vaccine acceptance.

**Fig. 3.**
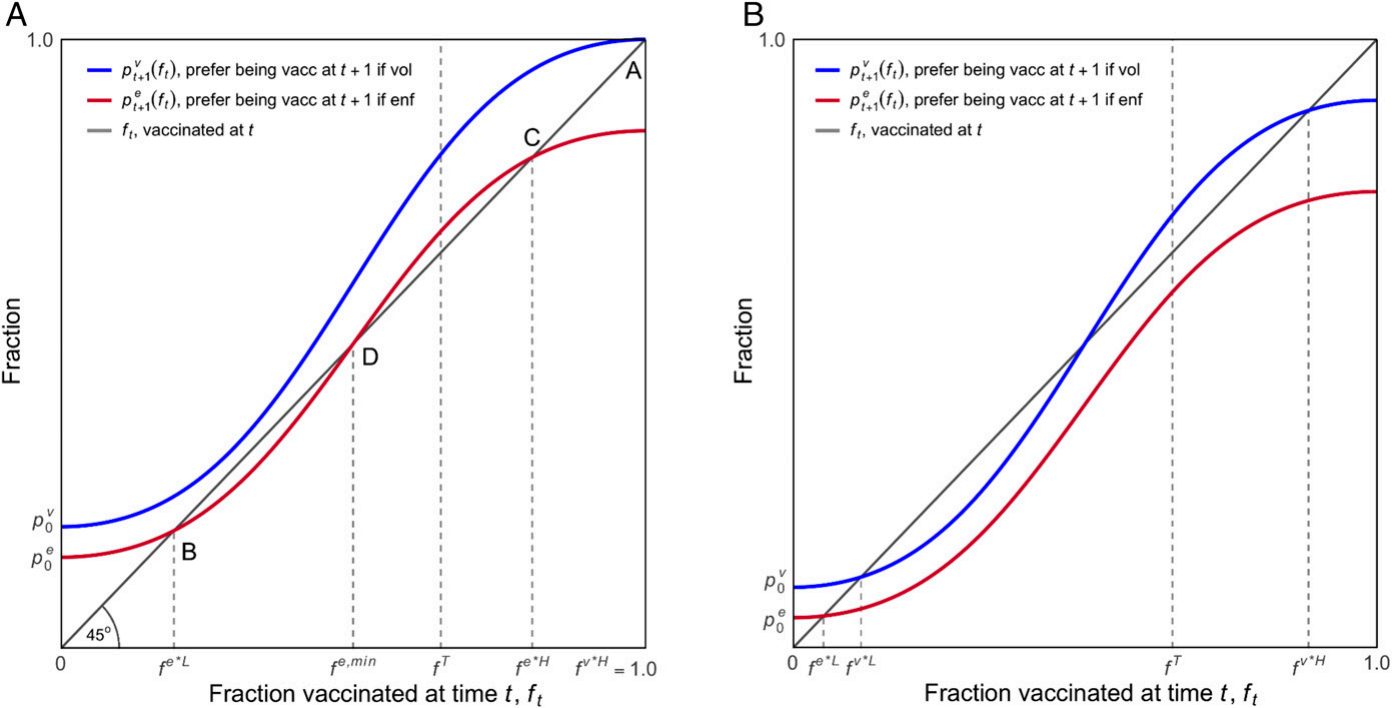
A model of the fractions preferring being vaccinated to not being vaccinated and the dynamics of vaccination under a voluntary and enforced policy. *A* is the “optimistic” scenario in which a recommended and voluntary vaccination policy surpasses the target level. *B* is the “pessimistic” scenario, as the target level of vaccinations will not be reached without enforcement.

We now turn to the model. At the beginning of each period, citizens know the total fraction that had been vaccinated by the end of the previous period and whether vaccinations are recommended or legally required. These two pieces of information determine the share of citizens that prefer being vaccinated to not being vaccinated. Then, in each period, any unvaccinated citizens who prefer being vaccinated receive the vaccine. The model is displayed in Fig. 3 and presented in more detail in *SI Appendix*.

The horizontal axis of Fig. 3 is the fraction of the population vaccinated at time *t*, with $f^{T}$ being the target level of vaccinations that the policy maker estimates is required to end the pandemic. The vertical axis refers to the next period, measuring two quantities. The first is the fraction of the population already vaccinated, which is identical to the *x*-axis (as given by the 45° gray line). The second, given the previous period’s fraction vaccinated, is the fraction who prefer the state of being vaccinated to not being vaccinated (irrespective of whether they are vaccinated.) This is shown in the S-shaped curves. The red curve indicates citizens’ preferences if vaccinations are legally mandated, and the blue curve if they are voluntary.

Similar S-shaped functions are commonly used in models of the diffusion of technological innovations, new products, or other novel practices and are often referred to as adoption curves (36, 46). These have been observed for a wide variety of novel adoptions including farmers switching to hybrid corn in the United States early in the last century and households purchasing radios, electric stoves, color TVs, microwaves, and computers more recently (47, 48).

The upward slope of the curves captures conformism (36): The more people have been vaccinated (in either case, enforced or voluntary), the more others will prefer being vaccinated. Conformism may arise because people develop a more positive assessment of vaccination if others have demonstrated their preference for being vaccinated or have been vaccinated without ill effects, or through mere exposure to more people who have been vaccinated.

The S-shape of conventional adoption curves represents a heterogeneous population with an approximately normally distributed threshold level of the number of other adopters that is just sufficient to induce an individual to view the novel practice favorably. The figure shows that some prefer being vaccinated even if no others are vaccinated. The first individuals being vaccinated convert just a few more from opposing vaccination to supporting it. The conformist effect increases as the population share of adopters increases. It later diminishes (the curve flattens) when the remaining nonadopters are a small minority strongly opposed to vaccination.

The model also works under alternative assumptions, for example, with linear adoption functions resulting from uniform distributions of resistances to vaccination. In this case, every additional vaccinated individual converts the same number of others to prefer being vaccinated, irrespective of the numbers already vaccinated (*SI Appendix*, Fig. S2).

The curves in the cases of voluntary and enforced vaccination differ in two ways.

First, based on our survey results, we assume that the number preferring being vaccinated for any given value of $f_{t}$ is greater if the treatment is voluntary rather than enforced. To convey some idea of the likely magnitudes, according to our second wave survey results (agreement levels 4 and 3 in Fig. 1*B*), the vertical distance between the two adoption curves should be about 25 percentage points—much greater than is shown in Fig. 3. (The *y*-axis intercepts differ by five percentage points and the maximum difference at $f_{t}$ = 1 is 15 percentage points.)

The second difference is that the voluntary curve is steeper. The slopes indicate the increased fraction of people who prefer vaccination if additional vaccinations are observed. The enforced curve is less steep because (as suggested above) the social signaling value of others having been vaccinated is less if they may have been vaccinated unwillingly.

The gray 45° line allows us to differentiate between the numbers already vaccinated and those who prefer being vaccinated. For example, in Fig. 3*A*, at point A, everyone is vaccinated and also prefers being vaccinated. For other values of $f_{t}$, the vertical distance between the blue curve and the 45° line is, for a voluntary vaccination regime, the fraction of the population that is not yet vaccinated but prefers being vaccinated to not being vaccinated. Using this information, we see that, if the adoption curve is above the 45° line, the fraction vaccinated will increase without enforcement.

Where the adoption curve and the 45° line intersect—for example, point B under an enforced vaccination regime—there are no unvaccinated individuals who will willingly (that is without enforcement) be vaccinated. If the adoption curve is below the 45° line, then some of those already vaccinated prefer not to have been, and there are no remaining willing but unvaccinated individuals. So, at each point from B to D, no increase in vaccinations will occur unless it is enforced.

In Fig. 3*A*, under an enforced vaccination policy, points B and C are “no regrets” equilibria in which all of those vaccinated prefer being vaccinated to not being vaccinated, even if some of those who are vaccinated at point C did not prefer being vaccinated at the time they received the vaccine. The intersection at D is a tipping point: If $f_{t}$ is somewhat greater than *f* ^*e*,min^, those as yet unvaccinated but preferring to be vaccinated will want to receive the vaccine, increasing $f_{t}$. If $f_{t}$ is somewhat less than *f* ^*e*,min^, then those vaccinated exceed those who prefer being vaccinated to not (some of those vaccinated would prefer not to have been vaccinated).

### Policy Implications.

Fig. 3*A* illustrates how the vaccination dynamic might unfold. If an enforced vaccination policy were adopted in the first period when none had previously been vaccinated, a fraction of the population $p_{0}^{e}$ would be willing to be vaccinated. They present themselves at a vaccination center, we assume, before any of the unwilling were required to be vaccinated. Once they have been vaccinated, according to the lower (enforced) adoption curve, additional members of the population would now voluntarily present themselves to be vaccinated, in response to which still more would come to prefer being vaccinated until the fraction *f* ^e*L^ has been vaccinated.

Beyond that fraction, to reach the target level, a fraction of the population equal to *f*^*e,min*^ − *f *^e*L^ would have to be vaccinated despite their preferring not to be vaccinated. If enforcement were effective, then, once the vaccination level exceeds *f *^*e,min*^, those preferring to be vaccinated would exceed those already vaccinated due to the conformism effect. So, while still mandatory, the fraction vaccinated would then increase without further enforcement, surpassing the target and reaching *f *^e*H^.

If the vaccination is recommended but not required by law, the “voluntary” adoption curve describes a quite different scenario in which initially $p_{0}^{v}$ are vaccinated, in response to which, in successive periods, vaccination spreads throughout the population eventually to *f*^v^^*^= 1 (point A). Fig. 3*B* describes a more pessimistic scenario in which voluntary vaccination reaches a fraction of the population less than the target level. In this case, effective enforcement is required to meet the target.

Our model and survey of course cannot determine whether the optimistic or pessimistic scenario (Fig. 3 *A* and *B*) is more likely. But these are empirical questions that can be answered, providing the policy maker with essential information about the wisdom of enforced versus voluntary policies. The policy maker who discovered that the relevant data are captured by Fig. 3*A*, for example, would know that a mandatory vaccination would be a costly policy error.

## Discussion

The COVID-19 pandemic has provided a rare lens for the study of the ways in which citizens’ preferences matter for the effectiveness of public policies and may be affected by the policies themselves. The socio-psychological aspects of policy effectiveness serving as the foundations of our model—conformism and crowding out—are not peculiar to COVID-19.

The implementation of any policy as a legally enforced requirement or a morally framed recommendation signals to citizens the nature of the relationship in which they are engaged: hierarchical if enforced or, alternatively, as members of a community perhaps subject to a social contract if voluntary (49, 50). As a result, policies adopted by a government (e.g., the uses of tax revenues) may alter the beliefs and preferences that shape people’s responses to policies, affecting essential prerequisites for good government such as the degree of tax compliance and the rule of law (51–53).

Beyond the pandemic, our findings apply to a large class of public policies—for example, encouraging lifestyle changes to address the climate emergency or promoting tolerance and respect in an ethnically heterogeneous society. These illustrate cases in which voluntary compliance is important because state capacities may be limited and because effectiveness depends critically on the ways that policies themselves may alter citizens’ beliefs and preferences.

Concerning COVID-19 policies, we do not know, of course, to what extent vaccination attitudes of Germans and their dynamics are informative about other populations. There is evidence that the effectiveness of some anti–COVID-19 policies differs across nations, while for others, this is not the case (54). Our survey, too, provides evidence for cultural differences. The fact that Germans born in the East responded differently to enforced and voluntary policies of vaccination (Fig. 2*A*) suggests that historical, cultural, and institutional differences have an important bearing on the choice of an effective vaccination strategy.

The aversion to enforcement we have observed here, however, is not peculiar to our panel; it has been documented in economic experiments in the United States, Italy, Switzerland, and both East and West Germany (17, 55–57). Moreover, we find that increased opposition to enforced vaccination over the two waves is common to both East and West Germans (*SI Appendix*, Table S4) despite their important historical differences.

The adoption curve we have used to illustrate the model is hypothetical, not estimated. But it is consistent with plausible psychological assumptions about the distribution of vaccine resistance and is also similar to many empirically estimated adoption curves. With sufficient research resources, it could, in principle, be estimated either from historical data on the take-up of voluntary vaccinations for other illnesses or collecting survey data prior to the launch of an antivirus vaccination campaign.

Subject to these caveats, the survey and model provide advice for policy makers.

First, mandating vaccination by law may have a substantial negative impact on voluntary compliance and may be unnecessary even if vaccine hesitancy is initially high. Given limited state capacities and citizen opposition, reaching the target in a timely manner by enforcement could be impossible and, in any case, might bear costs including heightened social conflict and further citizen alienation from government or professional elites. The result could be a negative cascade of public distrust fueling vaccine resistance, requiring more extensive enforcement and in turn further eroding public trust. However, we also show that if vaccine willingness is insufficient to induce cumulative increases in compliance, enforcement is unavoidable.

Second, an important anti–COVID-19 policy is to enhance public trust, possibly through greater transparency and accountability of political and professional elites (12). Active measures to enhance public trust could reduce the fraction of the population that would have to be required to be vaccinated unwillingly in order to surpass the target. We know from the panel that increasing public trust will shift upwards both adoption curves. This could transform the pessimistic scenario in Fig. 3*B* into the optimistic scenario in Fig. 3*A*, in which a policy recommending but not requiring vaccinations would be sufficient to surpass the policy maker’s target.

Third, convincing citizens that the vaccine is effective appears to be important, even if we cannot establish that the large estimated effect in Fig. 2*B* is causal. A person viscerally opposed to enforced vaccination may be more comfortable believing that vaccines are ineffective, so the causation could run in both directions. But as increasing numbers are vaccinated and infection incidence falls, it may be easier to disrupt a belief that the vaccine is ineffective, which in turn could undermine antivaccine sentiments.

Finally, our model suggests that even if the willing fraction is initially modest, reporting the prevalence of those already or willing to be vaccinated may be sufficient to induce a cascade of others to abandon their vaccination hesitancy. Conversely, media attention to those refusing the vaccine could also generate a conformist cascade of vaccine resistance.

## Materials and Methods

### The Questions.

To study the possibility that enforcement may crowd out civic values, it is essential not to confound social motives for an individual complying with a measure on the one hand with obedience to the law on the other. Therefore, our questions ask about the respondent’s attitude (“agree”) toward vaccination and not whether a person would comply with a legally imposed and enforced vaccination policy. Moreover, the voluntary option in our survey has a strong normative content (“strongly recommended”). Further explanation of why the questions were formulated this way are detailed in ref. 11.

### The Design.

To identify differential individual responses, all subjects were asked to state their agreement to get vaccinated in both cases: if it remains voluntary and if it is enforced. In a separate survey, we investigated the possibility of a demand effect due to asking a subject to answer both questions. We implemented a between-subjects design confronting respondents with only one alternative (either voluntary or enforced) and obtained very similar results. Altering the order of the alternatives in a within-subjects design did not affect average agreement with enforced or voluntary vaccination either.

To limit a potential spillover effect—a subject answering questions in a way to minimize cognitive inconsistency—the module containing the questions on agreement to get vaccinated and the module containing the questions about vaccine effectiveness and perceived restriction of freedom in case of enforcement were separated by a module unrelated to vaccination.

### The Panel.

The questions were embedded in an ad hoc online survey on COVID-19 initiated by the Cluster of Excellence “The Politics of Inequality” at the University of Konstanz.

Their predefined target sample size was 4,700 subjects in the first wave, and they aimed at 60% of those first-wave participants for the second wave. Participants were recruited from a commercial online access panel administered and remunerated by the survey provider respondi, which usually conducts market research. Membership of the respondi survey pool and participation in its surveys is voluntary and follows a double opt-in registration process. Participation is incentivized with tokens that can be exchanged for goods. Given this material incentive, people registered there are unlikely to have atypical intrinsic or social motivation relevant to the subject matter of the survey. This is important because otherwise, voluntary participation in the survey might create a sample bias in favor of voluntary policies.

The panel was implemented and run by the surveyLab at the University of Konstanz. The first wave was conducted from April 29 to May 8 and the second wave from October 28 to November 6, 2020. Our questions on agreement to get vaccinated in case it is voluntary or enforced were part of several modules on topics related to COVID-19. Invited participants self-selected into the online panel titled “Living in exceptional circumstances,” and subjects were not aware of the specific topic of any module (including ours) when agreeing to participate.

Before and after the modules, respondents answered a series of questions on socio-demographics and other controls. Basic demographics were mandatory to answer, in particular the questions concerning the sampling criteria. All other questions remained voluntary, and subjects were free to quit the survey at any time. In total, the first (second) wave of the panel contained 201 (203) variables and median response time was 14 (18) min.

### Participants.

Participants were required to be 18 y of age or older, German-speaking, and residents of Germany. The quota reflected the resident population in terms of (the marginal distributions of) age group, gender, education, and region. As East and West Germans have been shown to differ in their responses to enforcement (57) and as there are many fewer East Germans than West Germans, double quota for East Germany were used. All results reported in the paper and *SI Appendix* are based on unweighted observations. The mean age of the panel sample was 53 y (SD: 15 y), and 47% were female.

The following exclusion criteria were defined by the surveyLab: dropout during the survey, nonsense responses to open questions, speeders, and straightlining. Exclusions were performed by the surveyLab based on an independent standard quality check, without any involvement of the authors of this article. Moreover, we use list-wise exclusion of subjects with missing data in the variables used for the regressions. See *SI Appendix*, Table S1 for details.

This study was approved by the Ethics Committee of the University of Konstanz, IRB 20KN09-006. All subjects provided informed consent.

## Supplementary Material

Supplementary File

## Data Availability

The anonymized survey data and code files to replicate the results of the paper have been deposited at GESIS SowiDataNet datorium (German Data Archive for the Social Sciences) and are available at https://doi.org/10.7802/2272 (58).
