# Supplementary material for: Overcoming COVID-19 vaccination resistance when alternative policies affect the dynamics of conformism, social norms, and crowding out
Source: Proc Natl Acad Sci U S A. 2021 Jun 7;118(25):e2104912118. doi: 10.1073/pnas.2104912118 (PMC8237671; doi:10.1073/pnas.2104912118)
Supplement: Supplementary File [file pnas.2104912118.sapp.pdf]

PNAS
www.pnas.org

Supplementary Information for
Overcoming COVID-19 vaccination resistance when alternative policies affect the dynamics of conformism, social norms and crowding out

Katrin Schmelz and Samuel Bowles*

*Corresponding author
Email: katrin.schmelz@uni-konstanz.de

This PDF file includes:

Supplementary Text
- Main survey question on agreement to get vaccinated (original screenshot).
- Timeline of important news on COVID-19 in Germany before and during the two waves of the survey.
- The effect size of the change in trust in public institutions.
- Illustrations of adoption curves (including Fig. S1).
- Preference to be vaccinated under enforced and voluntary implementation in a model with conformism and crowding out (including Fig. S2).

Figures S3 to S4
- Fig. S3. Timing of the survey relative to the course of the COVID -19 pandemic in Germany.
- Fig. S4. Distributions of agreement to get vaccinated across the two waves of the survey if it is voluntary and enforced.

Tables S1 to S4
- Table S1. Number of participants in the panel, dropouts and exclusion criteria.
- Table S2. Explanation of all independent variables used in the regressions.
- Table S3. Predictors of agreement to get vaccinated in the second wave of the survey (linear OLS regressions, referring to Figs. 2A and 2B).
- Table S4. Predictors of the difference in agreement to get vaccinated between the two waves of the survey (linear OLS regressions, referring to Fig. 2C).

SI References

Supplementary Information Text

Main survey question on agreement to get vaccinated (original screenshot)

Einstellungen zu verschiedenen Maßnahmen

Wenn es einen zugelassenen Impfstoff gegen das Coronavirus gibt:

Inwieweit sind Sie einverstanden, sich selbst impfen zu lassen, wenn ...

	Überhaupt nicht einverstanden 0	1	2	3	Voll und ganz einverstanden 4
... die Impfung von der Regierung dringend empfohlen wird, aber freiwillig bleibt?	○	○	○	○	○
... die Impfung von der Regierung verpflichtend eingeführt und kontrolliert wird?	○	○	○	○	○

Zurück | Weiter

Timeline of important news on COVID-19 in Germany before and during the two waves of the survey

The timeline below shows that while the state of the pandemic differed substantially between the periods of the two surveys – cases were low and falling in May and high and rising in November – the information available to the public about the vaccines and the vaccination policies likely to be adopted by the German government were not substantially different.

All information reported below is taken from major German newspapers on the indicated dates.

News with respect to COVID-19 vaccines are highlighted in blue.

January 2020

27 January. First German infected with COVID-19.

February 2020

24 February. COVID-19 has arrived in Europe as an epidemic - serious situation in Italy.

March 2020

2 March.

- Recommendations: sneeze into the elbow, wash your hands regularly, avoid handshakes.

10 March. Recommendation: cancellation of all major events with more than 1,000 participants.

16 March. Press conference by Chancellor Merkel on anti-COVID-19 lockdown measures:

- Schools and day-care centers are already closed in most of the federal states, others will follow.
- Shops are to close – except for supermarkets, pharmacies, drugstores, petrol stations and hairdressers.
- Restaurants may only open between 6am and 6pm.
- Places of worship, playgrounds, sports facilities, bars, clubs, theatres, cinemas, concert halls and museums will be completely closed.
- Restrictions on travel, borders are closed.

18 March.

- Historic TV address of Chancellor Merkel. In an urgent appeal, she calls on the population to act in solidarity and responsibility. “Social contacts must be minimized.” “This is serious. Take it seriously, too.”
- EU imposes entry ban.
- Robert-Koch-Institute (RKI): coronavirus vaccine realistic by spring 2021.

20 March. Bavaria imposes state-wide curfew.

22 March.

- Federal and state governments agree on strict restrictions on exit and contact. Citizens may only be in public areas with a maximum of one person who does not live in the same household and must keep at least 1.5m distance.
- Restaurants and pubs may only offer take-away food. Hairdressers must close.

28 March. Infection Protection Act comes into force (i.e., the government is entitled to restrict fundamental rights).

April 2020

1 April.

- The nationwide contact restrictions are extended until 19 April. People should generally refrain from private travel and visits - including those by relatives.

15 April.

- The severe restrictions on contact will be extended until 3 May.
- Stepwise reopening of schools on May 4.
- Restaurants, bars and pubs are to remain closed as before.
- Major events will also remain prohibited until at least 31 August.

16 April. Government recommends to wear community masks when shopping and in public transport.

17 April. Germany survived the first wave of Covid-19 well, gradually returning to normality.

20 April. First cautious relaxations of the anti-Covid-19 measures come into force.

22 April. Paul-Ehrlich-Institute has approved the first clinical trial of a corona vaccine to be tested in Germany.

27 April. Wearing face masks is mandatory in all federal states for shopping and public transport.

SURVEY WAVE 1 STARTS ON 29 APRIL

29 April.

- First test subjects of German vaccination study have been injected.
- Minister of Health Spahn: enforced vaccination will not be necessary, voluntary willingness to get vaccinated is sufficient.

30 April. Chancellor Merkel is consulting with the heads of the federal states on how to proceed:

- Contact restrictions remain in force for the time being. Citizens are to keep a minimum distance of 1.5 meters in public and only stay there alone, with another person not living in the household or with members of their own household.
- Playgrounds are to be permitted again under certain conditions.
- Community worship services should be allowed again with rules on distance and hygiene.
- Schools and daycare centers: no changes, federal and state governments want to discuss this in more detail on 6 May.
- Restaurants, hotels and cafés will remain closed.
- No changes for the time being with respect to store openings.

May 2020

2 May.

- Some federal states relax some measures, contrary to the federal and state agreements from 30 April.
- Demonstrations against the corona restrictions.

3 May. Minister of Health Spahn thinks that Germany will stick to voluntary vaccinations. Some other prominent political figures, e.g. Bavaria's Prime Minister Söder, express that they would favor enforcement.

4 May. Hairdressers reopen.

5 May. RKI head Lothar Wieler pleads for flexible relaxations of the measures in Germany, depending on the local incidence of infections.

6 May. The federal and state governments have agreed on further relaxations, though contact restrictions remain largely in place:

- Families from two different households can meet.
- Schools: gradual reopening.
- Daycare: remain closed.
- Hotels and restaurants: gradual reopening, subject to hygiene and distancing conditions.
- Shops: all shops can open but have to meet hygiene and distancing requirements. Wearing masks is mandatory.
- Districts with more than 50 infections per 100,000 inhabitants within the last 7 days will have to return to stricter restrictions immediately.

8 May.

- Three districts already break the negotiated upper limit for new infections.
- Growing resistance against the corona measures. Critics say that the restrictions of basic rights are too severe.
- Many fear a compulsory COVID-19 vaccination - for a vaccine that does not yet exist.

SURVEY WAVE 1 ENDS ON 8 MAY

16 May. RKI and government confirm that relying on citizens' sense of responsibility, vaccinations will be voluntary.

August 2021

11 August. Putin announces Russia's approval of the world's first COVID-19 vaccine *Sputnik* to the media.

September 2020

15 September. Health Minister Spahn has reiterated that COVID-19 vaccination will not be compulsory.

17 September. Many Germans hesitant – will voluntary vaccination work?

24 September. According to the German Standing Committee on Vaccination (STIKO), it is unclear whether enforcement of COVID-19 vaccinations will be beneficial. Someone who absolutely does not want to be vaccinated will always find ways to get around it.

October 2020

1 October.

- *CureVac* expects vaccine in the first half of 2021. Second phase of clinical trial started this week.
- Minister of Health Spahn: Prioritized vaccination may be needed, such that, for example, health care workers and at-risk groups get the vaccine first.

2 October. Research is being conducted on many possible COVID-19 vaccines worldwide. A German company also has a promising candidate in the decisive test phase. When will the vaccination come?

5 October. Experts at the RKI are dampening hopes for a quick return to normality after a vaccine is admitted. This is because it will initially only be available in limited quantities.

6 October.

- *AstraZeneca* announces to provide vaccine data from trial series by the end of the year.
- European Medicines Agency (EMA) approval process for *BioNTech/Pfizer* vaccine starts.

7 October. The U.S. Food and Drug Administration has tightened the requirements for emergency approval of a COVID-19 vaccine - apparently against the will of the White House.

8 October.
- The number of infections in Germany is increasing dramatically and almost reaches the mark of 4,000 new cases in one day. Alarming thresholds are exceeded in Berlin and Frankfurt.
- Research Minister Anja Karliczek expects widespread COVID-19 vaccination to be possible from mid-2021. Currently, three companies are receiving federal funding for vaccine development.

10 October. Although Chinese vaccine candidates are still in the final testing phase, hundreds of thousands of Chinese are already being vaccinated. It is still unclear how safe and effective the Chinese vaccines actually are.

12 October.
- One district exceeds the critical mark of 50 new infections per 100,000 inhabitants within seven days.
- Federal Health Minister Spahn expects that vaccinations in Germany can begin in the first quarter of 2021. People with pre-existing health conditions, the elderly and health and care workers will be targeted first.

14 October. The federal and state governments agree on new containment policies in hotspots:
- In regions with 50 or more new infections per 100,000 inhabitants within seven days, private parties are to be limited to a maximum of ten people and two households. There is to be an 11 p.m. curfew for restaurants.
- In regions with 35 new infections per 100,000 inhabitants within seven days, the mask requirement is also to apply where people gather more closely or for longer periods.

15 October.
- The number of new infections in Germany rises to over 6,000 within a day.
- Reactions to the federal-state resolutions on infection control are polarized.
- Bavaria issues its own, stricter regulations.
- Courts in two federal states overturn a controversial ban on accommodation for guests from high-risk regions within Germany, while other state governments suspend it.

17 October.
- In view of the sharp rise in new infections, Chancellor Merkel is calling on the population to reduce contacts as far as possible.
- *BioNTech/Pfizer* has already started mass production of a vaccine.
- *Pfizer* will apply for emergency approval in the U.S. by the end of November.

13-18 October. Reports of fake news claiming that vaccination would be compulsory and fact checks that this is not true.

19 October. The first district in Germany has again very strict exit and contact restrictions.

20 October. *Moderna* says its vaccine could receive U.S. emergency approval in December.

23 October.
- German Federal Ministry of Health reaffirms expected COVID-19 vaccination start in early 2021. Previously, there have been media reports about a possible vaccination start this year.
- Health Minister Spahn: there will be no compulsory vaccination.

25 October. COVID-19 opponents are mobilizing not only on social media, but also with flyers containing misleading and false claims.

26 October. *AstraZeneca*'s vaccine appears to elicit a "robust immune response" among the elderly. Results will be published soon.

SURVEY WAVE 2 STARTS ON 28 OCTOBER

28 October. Second lockdown "light" announced in Germany, lasting for the entire November:
- Restaurants and houses of culture close, no tourism.
- Contact restrictions of at most 10 people or two households.
- Schools and daycare remain open.
- Commerce and business will keep running.

29 October.
- German Standing Committee on Vaccination (STIKO) announces that Germany will not be fully vaccinated until 2022.
- According to an article in The Lancet by the chair of the U.K. Vaccine Task Force, upcoming vaccines "probably won't be perfect" and "may not work for everyone".

30 October. EU spreads optimism, is the vaccine coming soon? European Parliament is discussing December/January as start dates for vaccinations. Health professionals are to be vaccinated first. But the first vaccines may not fully protect everyone.

31 October. Paul Ehrlich Institute expects first vaccine approvals in early 2021. However, the approval of a vaccine does not mean that it will be immediately available to the entire population.

November 2020

1 November.
- A suggestion for the national vaccination strategy will be presented in the coming week. It will address an ethically sensitive question: who comes first, who comes last?
- Nearly one in ten German health departments complains of being overwhelmed.

2 November. Lockdown "light" starts.

3 November.
- The vaccine of the Germany-based company *CureVac* has successfully passed the first clinical test phase. The 250 subjects showed a responsive immune response and good tolerability.

5 November. Despite tight contact restrictions, COVID-19 infections in Europe continue to rise rapidly.

6 November.
- German government classifies almost all of Europe as a risk area. Hospitals in Germany are preparing contingency plans for the treatment of Covid-19 patients.
- Mutation of SARS-CoV-2 found on a mink farm in Denmark, concern that the vaccines developed so far might not work against mutations.

SURVEY WAVE 2 ENDS ON 6 NOVEMBER

9 November.
- *BioNTech/Pfizer* announce that a vaccine will be available soon, 90% success rate.
- Chancellor Merkel announces that the lockdown policies will become more stringent instead of relaxed.

16 November. *Moderna* announces a new vaccine with 94% effectiveness.

The effect size of the change in trust in public institutions

We regressed the change between the two waves of the survey in support for enforced vaccinations (mean = 0.513; sd = 1.524) on the change in trust in public institutions where both are measured in Likert points (instead of standard deviation units), along with other explanatory variables shown in table S4. The coefficient of the change in public trust variable is 0.192 (CI: 0.120; 0.265). So, a hypothetical one Likert point increase in public trust on average would have eliminated 37.5 percent (0.192 / 0.513) of the decline in support for enforced vaccination.

Illustrations of adoption curves

Illustrations of adoption curves for the case of hybrid corn in the U.S. appear in Fig. S1 with the extent of adoption as a function of the date (not the number of previous adopters).

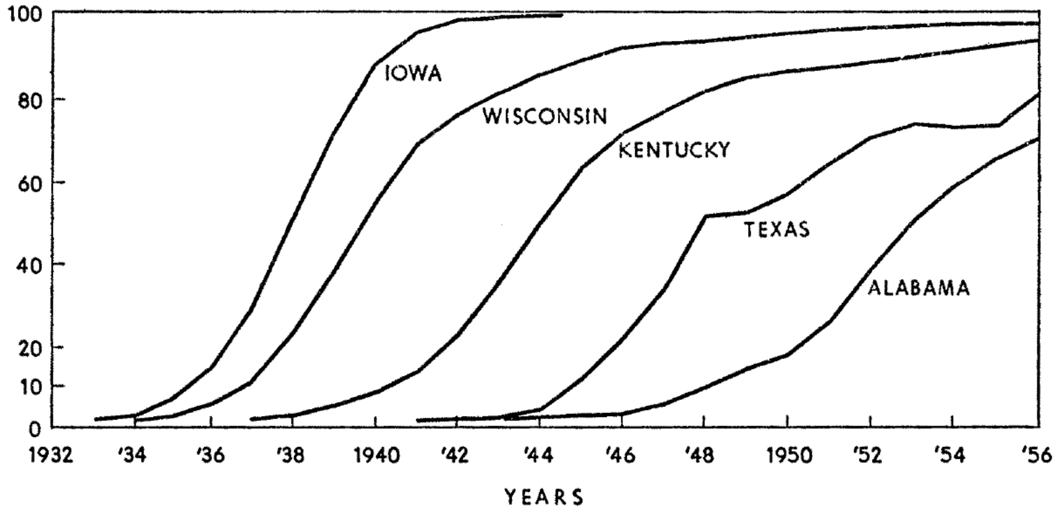

Fig. S1. Fraction of total corn acreage planted with hybrid seed, U.S. states 1932-1952 (1).

Preference to be vaccinated under enforced and voluntary regimes in a model with conformism and crowding out

We model how the fraction of people who prefer the state of being vaccinated to the state of not being vaccinated (independently of whether they are now vaccinated) at time t + 1, that is p_{t+1}, depends on the fraction already vaccinated in time t, $p_{t+1}(f_t)$. As explained in the main text, we consider a heterogeneous population in which people have varying resistances to being vaccinated, ranging from those who prefer to be vaccinated even if no others have been vaccinated to those who prefer to not be vaccinated unless at least f others have been vaccinated.

To explore the model's structure, we begin with a simple case in which $p_{t+1} = p_0 + \alpha f_t$ as shown in Fig. S2. This linear adoption curve means that irrespective of the numbers already vaccinated, every additional vaccinated individual converts the same number of others to pro-vaccination.

Consider the adoption function shown by the solid green line. If in time t there are fewer than f_t^* vaccinated then those preferring vaccination to not being vaccinated in the next period will exceed those already vaccinated, and the numbers of those being willingly vaccinated will increase. An upward shift in the adoption function – due to, say, an increase in public trust – would result in a proportionally greater increase in those being willingly vaccinated from f_t^* to $f_t^{*\Delta}$ as illustrated by the dashed green line.

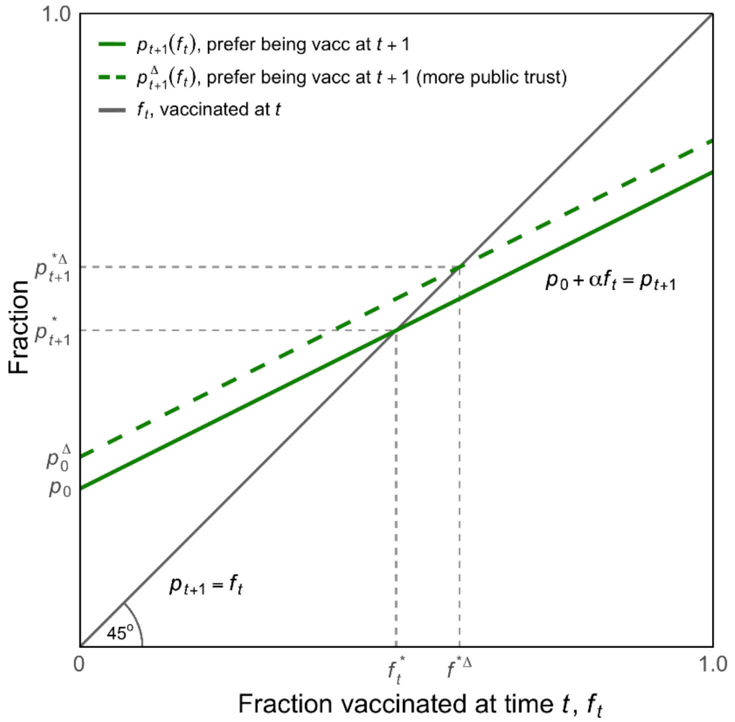

Fig. S2. Illustrating the model of vaccination acceptance with a linear adoption curve.

Reflecting the adoption curves illustrated in Fig. S1, we use the following adoption function:

$$(1) \quad p_{t+1}^{i} = p_0^{i} + \alpha^{i} \frac{f_t^{\mu}}{f_t^{\mu} + (1 - f_t)^{\mu}}$$

where the exponent $\mu > 1$ gives the S-shape shown in Fig. 3 (higher values of μ give a more pronounced curvature). The superscript *i* is either *e* or *v,* referring to the adoption function in the case where vaccination is legally required (enforced) or only recommended (voluntary).

Our model is designed to capture four effects. The first, based on our survey findings, is a *public trust effect*, according to which the adoption curve will be higher the greater is the trust in the institutions governing a population and providing it with information about the pandemic. While changes in the level of trust could affect the function in more complex ways, it is natural to associate changes in public trust with differences in the constant, p_0^{i}.

The second is a *conformism effect* on citizens' preferences, which is captured by the function $p_{t+1}(f_t)$ being increasing in its argument.

The third is a *crowding out effect* (enforcement crowding out voluntary agreement) which we model as $p_0^{v} > p_0^{e}$ indicating that more citizens are willing to be vaccinated voluntarily than under enforcement. This is consistent with the data shown in Figs. 1A and 1B.

Fourth, we include an *effect of enforcement on conformism* $\alpha^{v} > \alpha^{e}$, capturing the idea that citizens are more likely to copy the behaviors of fellow citizens if they believe that their fellow citizens' actions have been freely chosen rather than enforced, because a free choice is a stronger signal of a person's attitude than an enforced choice. This fourth effect means that conformism and voluntariness of the vaccination regime are complements: the positive effect on vaccine acceptance of additional people having already been vaccinated will be greater under a voluntary than an enforced regime (the voluntary curve in Figure 3 is steeper). Correspondingly, the positive effect of a regime being voluntary will be greater the more people have already been vaccinated (the vertical distance between the two curves is greatest at $f_t = 1$).

Equation (1) describes an irreversible dynamical system (people cannot undo their vaccination). Thus, under either policy ($i \in (e, v)$), the change in those who will be vaccinated without being enforced is:

$$(2) \quad \Delta p_{t+1}^{i} = 0 \text{ if } p_{t+1}^{i} \leq f_t$$

$$(3) \quad \Delta p_{t+1}^{i} > 0 \text{ if } p_{t+1}^{i} > f_t$$

From Equations (2) and (3) we see that the stationary fractions vaccinated under a voluntary regime in Fig. 3A are $f_t = f^{v*H} = 1.0,$ and under enforcement are $f_t = f^{e*H}$ as well as (because of the irreversibility property) all of the fractions from $f_t = f^{e*L}$ to $f_t = f^{e,\min}$.
If we have $f_t > p_{t+1}^{i}(f_t)$ then some of those already vaccinated at time *t* would, in time *t+1,* prefer not to have been vaccinated. This could be the case had they been vaccinated under an enforced vaccination regime, or if their public trust had diminished, shifting downwards the adoption curve.

The two scenarios shown in Fig. 3 illustrate conditions under which enforcement may be avoided (A) or may be required (B). In the second, "pessimistic" scenario, the target level cannot be attained through voluntary vaccination unless public policy can shift up the voluntary adoption

curve. Otherwise, in order to reach the target, vaccination would have to be enforced. The fraction of the population that would have to be vaccinated against their will would be $f^T - f^{e*L}$. This number would be reduced, possibly to zero, by an increase in public trust because it would shift upwards the adoption function pertaining to an enforced policy, implying that even under enforcement, more people would get vaccinated willingly.

The values that generated the adoption curves in Fig. 3 are as follows:

- curvature of the adoption function, μ, 1.9;
- degree of conformism, α^i for voluntary 0.8 and for enforced, 0.9;
- constant in the adoption function, p_0^i, 0.2 and 0.15 for voluntary and enforced respectively in panel A; and 0.1 and 0.05 respectively in panel B.

We chose parameters such that in panel A, $p^v(1) = 1$, so that all prefer being vaccinated when all have been vaccinated. It could be that the adoption curve would be downward sloping because if all others are vaccinated the last unvaccinated citizen might have no private incentive to be vaccinated (2). This would be true, for example, if vaccinations were motivated entirely by individual self-interest and vaccines were commonly known to be 100 percent effective, neither of which is likely to true for COVID-19 vaccines. But in any case, the parameterization in Fig. 3A that allows for universal voluntary vaccination is not of substantive importance.

Equation (1) together with the parameters mentioned above imply that the vertical distance between the two adoption curves in Fig. 3 (both panels) is substantially less than the difference shown in Fig. 1B between the empirically estimated fraction preferring to be vaccinated under a voluntary as opposed to an enforced regime. This is true even for the maximum value of $p^v - p^e$ (which obtains when $f_t = 1$) which is $p^v(1) - p^e(1) = 0.15$. (When $f_t = 1$, the fraction in Equation (1) equals 1, so $p^v(1) - p^e(1) = p_0^v - p_0^e + \alpha^v - \alpha^e$.) The empirically estimated difference in the fraction supporting vaccination when voluntary versus when enforced exceeds 0.2 in the second wave of the survey. We are thus reasonably sure that Fig. 3 does not overstate the degree to which enforcement may crowd out willingness to be vaccinated.

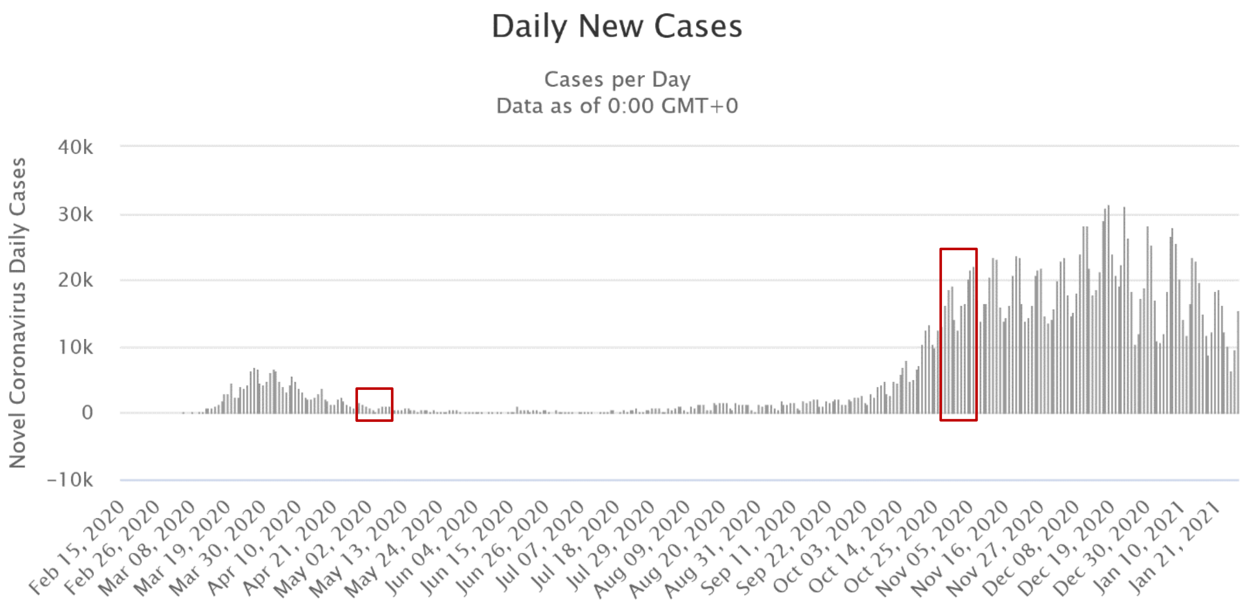

Fig. S3. Timing of the survey relative to the course of the COVID-19 pandemic in Germany. The red frames indicate the time frames of the first and the second wave of our survey. The chart is taken from worldometers.org (4).

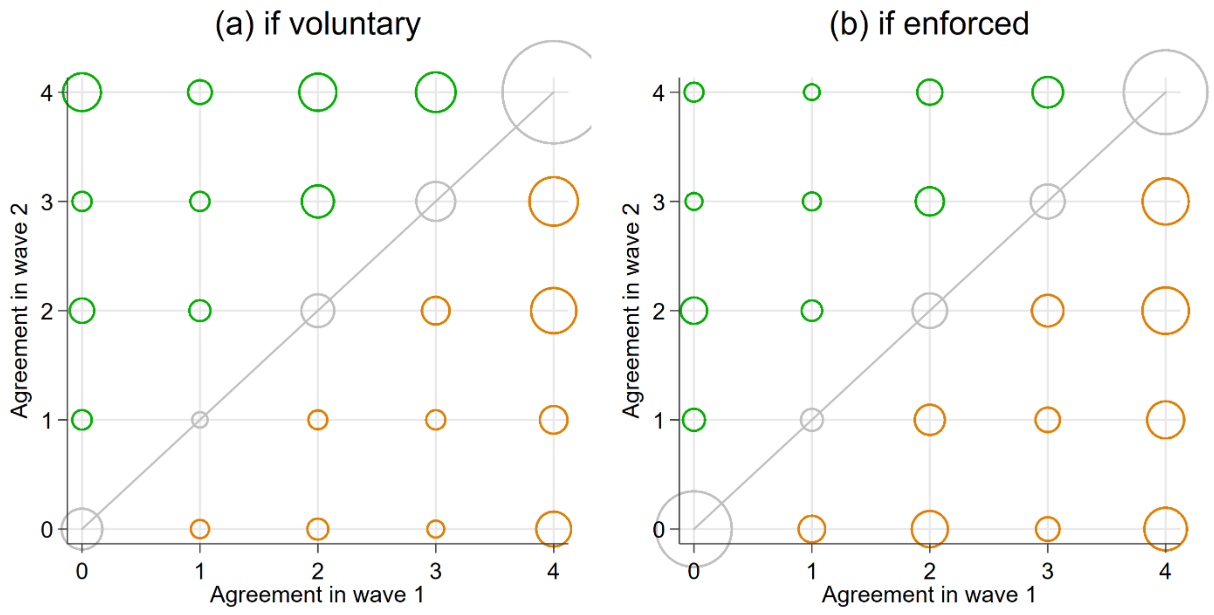

Fig. S4. Distributions of agreement to get vaccinated across the two waves of the survey if it is voluntary (panel a) and enforced (panel b). The sample size is n = 2,653. The size of a bubble increases with the frequency of respondents. Bubbles on the grey line represent answers which remain unchanged between the first and the second wave, i.e., an individual's agreement in wave 1 equals her agreement in wave 2. Green bubbles above the diagonal represent individuals who increase their agreement in the second wave. Orange bubbles below the diagonal refer to individuals whose agreement is lower in the second than in the first wave.

Table S1. Number of participants in the panel, dropouts and exclusion criteria. Exclusions according to the upper eight criteria were performed by the surveyLab, based on an independent quality check.

	Number of participants
Survey wave 2 started	3,264
Dropout at the very beginning of the survey (compulsory socio-demographic information incomplete)	132
Survey not completed	187
Year of birth > 2002 (no technical restriction in wave 2)	3
Very high number of missing answers	17
Nonsense responses to open questions	5
Speeders who completed the survey in less than 7.8 min (corresponds to <40% of median interview duration)	127
Straightlining (same responses across various question blocks)	32
Failed quality check of wave 1	78
Missing answer on at least one of the four questions on agreement to get vaccinated (2 waves x 2 policies)	30
**Basis for the article (Fig. 1)**	**2,653**
Reported gender differs between the two waves	16
Missing values for other variables used in the regressions	81 to 92
**Used in the regressions (Fig. 2)**	**2,545 to 2,556**

Table S2. Explanation of all independent variables used in the regressions. Q1 refers to the first quartile, Q2 refers to the second quartile (median), and Q3 refers to the third quartile. All variables are used from the second wave of the survey (except for the two variables on differences between the two waves).

Variable	Description and remarks	Survey question	Distribution
Public_trust	Average of 5 measures of trust in public institutions: trust in federal government, trust in state government, trust in science, trust in media, and trust in government's truthful information about COVID-19.<br><br>The 5 measures are highly correlated (mean correlation = 0.68, min = 0.60). In a PCA, the first component explains 74% of the variance, suggesting one underlying component.	Here you can now see a number of public bodies and institutions. How much confidence do you generally have in them?<br>Federal government<br>State government<br>Experts from science<br>Media<br>(7-point Likert scale ranging from 1 "no confidence at all" to 7 "a great deal of confidence") (5)<br><br>How factually truthful do you think your country's government has been about the coronavirus outbreak? (very untruthful – somewhat untruthful - neither nor - somewhat truthful - very truthful) (6)<br><br>The combined variable ranges from 1 (lowest trust) to 6.6 (highest trust).	Mean=3.99, SD=1.45, Q1=3, Q2=4.2, Q3=5.2, missing: n=0.
Public_trust_diff	Difference in *Public_trust* between the two waves of the survey (wave 1 – wave 2).		Mean=0.01, SD=0.82, Q1=-0.40, Q2=0, Q3=0.40, missing: n=0.
Vaccine_effective	Survey question only included in wave 2.	What do you think: How effective are the following measures (if most people comply) in containing the spread of the coronavirus? … vaccination against the coronavirus (5-point Likert scale ranging from 0 "not at all effective" to 4 "highly effective")	Mean=2.80, SD=1.31, Q1=2, Q2=3, Q3=4, missing: n=4.
Vaccine_freedom	Survey question only included in wave 2.	Assume that the following anti-COVID-19 measures are mandatory and checked.<br>To what extent do you feel this restricts your freedom? … vaccination against the coronavirus (5-point Likert scale ranging from 0 "not at all restricted in my freedom" to 4 "absolutely restricted in my freedom")	Mean=1.60, SD=1.61, Q1=0, Q2=1, Q3=3, missing: n=8.
Altruism	Proxy for altruism.	I feel a moral obligation to help people in need. (7-point Likert scale ranging from 1 "do not agree at all" to 7 "agree completely")	Mean=4.86, SD=1.60, Q1=4, Q2=5, Q3=6, missing: n=6.

Age	In years at the time of the survey, computed from year of birth.		Mean=52.77 years, SD=14.62, Q1=42, Q2=55, Q3=64.
Female	Dummy variable which takes the value one for female and zero for male.		47% female, 53% male.
High_education	Dummy for high levels of schooling. It takes the value zero for low and intermediate levels of schooling.	What is your highest school leaving certificate?<br>(subjects choose from a list of options)	33% high education, 67% middle or low education.
East_childhood	Dummy variable taking the value one if a person spent her or his childhood in a federal state of East Germany. It takes the value zero for West Germans and is missing otherwise.	In which federal state did you spend most of your childhood?	30% East, 68% West, 2% missing (n=42).
Household_income	Subjects could choose to answer an open or a categorical question on monthly net household income (in euros). The variable is constructed by combining the answers to both questions (done by the *surveyLab*).	6 categories where 1 refers to <900 euros and 6 refers to >=6000 euros.	Mean=3.28, SD=1.36, Q1=2, Q2=3, Q3=4, missing: n=11.
Single_household	Dummy which indicates whether the person lives alone. It takes the value zero if more than one person live in the respondent's household.	How many people currently live in your household, including yourself?	32% single households, n=0 missing (answer was mandatory).
Survey_day	Refers to the 10 days of survey participation in wave 2.		Mean=5.12, SD=2.30, Q1=3, Q2=6, Q3=7.
Cov19_risk_group	Dummy indicating whether a person would be at high risk when infected with COVID-19.	Do you belong to the COVID-19 risk group due to pre-existing health issues? (yes/no)	46% risk group, 53% not risk group, 1% missing (n=19).
TotalCov19_per100k	Total (cumulative) number of COVID-19 cases per 100,000 inhabitants registered in the federal state of the participant's residence in wave 2 at the day prior to survey participation, as published by the RKI (Robert-Koch-Institute, German government's central scientific institution responsible for diseases, (7)).		Mean=599, SD=219, Q1=413, Q2=680, Q3=777, missing: n=0.
TotalCov19_per100k_diff	Increase in *TotalCov19_per100k* from wave 1 to wave 2.		Mean=422, SD=167, Q1=292, Q2=451, Q3=552, missing: n=0.
Cov19_critical_locally	Results from Tables S4 and S5 are shown in Figs. 2A and 2B. Survey question only included in wave 2.	How critical do you think the COVID-19 situation currently is in your region? (9-point Likert scale ranging from 1 "not critical at all" to 9 "highly critical")	Mean=5.76, SD=2.24, Q1=4, Q2=6, Q3=7, missing: n=4.

Table S3. Predictors of agreement to get vaccinated in the second wave of the survey. Linear OLS regressions, referring to Fig. 2A (models 1 and 3) and Fig. 2B (models 2 and 4). All dependent and independent variables are standardized (except for dummies). See Table S2 for explanations of the independent variables.

	**Agreement to get vaccinated in wave 2**			
	if voluntary		if enforced	
	(1)	(2)	(3)	(4)
Public_trust	0.188***	0.105***	0.350***	0.095***
	(0.022)	(0.024)	(0.020)	(0.018)
Vaccine_effective		0.179***		0.338***
		(0.027)		(0.020)
Vaccine_freedom		-0.011		-0.351***
		(0.024)		(0.018)
Altruism	0.058***	0.059***	-0.037**	-0.027*
	(0.020)	(0.020)	(0.018)	(0.015)
Age	0.078***	0.066***	0.109***	0.034**
	(0.022)	(0.022)	(0.020)	(0.016)
Female	-0.036	0.012	-0.201***	-0.043
	(0.039)	(0.039)	(0.035)	(0.029)
High_education	0.158***	0.133***	0.074*	0.040
	(0.044)	(0.044)	(0.040)	(0.033)
East_childhood	-0.086*	-0.083*	0.094**	0.087**
	(0.051)	(0.050)	(0.045)	(0.037)
Household_income	0.028	0.026	-0.035*	-0.031*
	(0.022)	(0.022)	(0.020)	(0.016)
Single_household	0.025	0.031	-0.129***	-0.085**
	(0.045)	(0.045)	(0.041)	(0.033)
Survey_day	0.013	0.019	-0.003	-0.001
	(0.020)	(0.020)	(0.018)	(0.014)
Cov19_risk_group	0.014	-0.017	0.117***	0.026
	(0.043)	(0.043)	(0.039)	(0.032)
TotalCov19_per100k	-0.020	-0.018	-0.014	-0.006
	(0.024)	(0.024)	(0.021)	(0.018)
Cov19_ critical_locally	0.051**	0.017	0.190***	0.065***
	(0.022)	(0.023)	(0.020)	(0.017)
Constant	-0.022	-0.024	0.028	-0.005
	(0.042)	(0.041)	(0.037)	(0.031)
Observations	2,556	2,545	2,556	2,545
R-squared	0.077	0.099	0.250	0.503

Standard errors in parentheses
*** p<0.01, ** p<0.05, * p<0.1

Table S4. Predictors of the difference in agreement to get vaccinated between the two waves of the survey. Linear OLS regressions, referring to Fig. 2C (models 2 and 4). All dependent and independent variables are standardized (except for dummies). See Table S2 for explanations of the independent variables. (Note: The comparison in the main text of the cross-section and panel-estimated effects of public trust are based on models 1 and 3 of this and the previous table, rather than models 2 and 4, because measures on vaccine attitudes are not standard in the cross-section data sets which form the basis of most estimates of the effects of public trust.)

	Difference in agreement to get vaccinated (wave 1 - wave 2)			
	if voluntary		if enforced	
	(1)	(2)	(3)	(4)
Public_trust_diff	0.056***	0.057***	0.106***	0.103***
	(0.020)	(0.020)	(0.020)	(0.020)
Vaccine_effective		0.011		-0.019
		(0.026)		(0.026)
Vaccine_freedom		-0.010		0.106***
		(0.025)		(0.025)
Altruism	-0.048**	-0.047**	0.010	0.012
	(0.020)	(0.020)	(0.020)	(0.020)
Age	0.005	0.000	0.005	0.026
	(0.023)	(0.023)	(0.023)	(0.023)
Female	-0.018	-0.014	0.098**	0.066
	(0.041)	(0.041)	(0.040)	(0.041)
High_education	-0.031	-0.039	-0.025	-0.016
	(0.046)	(0.046)	(0.045)	(0.046)
East_childhood	0.072	0.067	0.031	0.023
	(0.049)	(0.050)	(0.049)	(0.049)
Household_income	0.000	0.005	0.051**	0.051**
	(0.023)	(0.023)	(0.022)	(0.022)
Single_household	-0.008	0.004	0.040	0.035
	(0.047)	(0.047)	(0.047)	(0.046)
Cov19_risk_group	-0.021	-0.022	-0.003	0.008
	(0.044)	(0.045)	(0.044)	(0.044)
TotalCov19_per100k_diff	-0.009	-0.010	-0.000	-0.006
	(0.023)	(0.023)	(0.023)	(0.023)
Cov19_ critical_locally	0.029	0.021	0.002	0.040*
	(0.021)	(0.023)	(0.021)	(0.023)
Constant	0.007	0.006	-0.054	-0.042
	(0.043)	(0.043)	(0.042)	(0.042)
Observations	2,556	2,545	2,556	2,545
R-squared	0.007	0.007	0.015	0.027

Standard errors in parentheses
*** p<0.01, ** p<0.05, * p<0.1

SI References

1. Z. Griliches, Hybrid Corn: An exploration of the economics of technological change. *Econometrica* **25**, 501-522 (1957).
2. P.-Y. Geoffard, T. Philipson, Disease eradication: Private versus public vaccination. *American Economic Review* **87**, 222-230 (1997).
3. P. Young, The spread of innovations by social learning. *University of Oxford Working Paper* (2006).
4. Worldometer. *Worldometers coronavirus*, https://www.worldometers.info/coronavirus/country/germany/ (2021), accessed: January 30, 2021.
5. R. Inglehart, C. Haerpfer, A. Moreno, C. Welzel, K. Kizilova, J. Diez-Medrano, M. Lagos, P. Norris, E. Ponarin & B. Puranen et al. (eds.). *World Values Survey: Round Six*, http://www.worldvaluessurvey.org/WVSDocumentationWV6.jsp (2014), accessed: June 15, 2020.
6. T. Fetzer, Witte, M., Hensel, L., Jachimowicz, J.M., Haushofer, J., Ivchenko, A., Caria, C., Reutskaja, E., Roth, C., Fiorin, F., Gomez, M., Kraft-Todd, G., Goetz, F., & Yoeli, E. *Global Behaviors and Perceptions in the COVID-19 Pandemic*, https://covid19-survey.org/ (2020), accessed: April 7, 2020.
7. Robert-Koch-Institut. *RKI Corona Bundesländer*, https://www.rki.de/DE/Content/InfAZ/N/Neuartiges_Coronavirus/Situationsberichte/Gesamt.html (2020), accessed: April/May and October/November, 2020.
